# Protein Paradox: Protein Quality Based on Amino Acids Composition is Poorly Associated with Health Outcomes

**DOI:** 10.1007/s13668-026-00754-4

**Published:** 2026-04-21

**Authors:** Miguel López-Moreno, Gabriel Barrantes-Espinola, Joel C. Craddock

**Affiliations:** 1 Institute of Health and Sport Sciences, Faculty of Health Sciences, Universidad Francisco de Vitoria, Madrid, Spain; 2 Nutrición y dietética, Facultad de Ciencias de la Salud, Universidad San Ignacio de Loyola, Lima, Perú; 3 School of Medical, Indigenous and Health Sciences, Faculty of Science, Medicine, Health University of Wollongong, Wollongong, Australia

**Keywords:** Protein quality, Food matrix, Chronic disease, Plant protein, DIAAS

## Abstract

**Purpose of Review:**

We examine the role and limitations of protein quality metrics, with a particular focus on the Digestible Indispensable Amino Acid Score (DIAAS), when applied beyond the assessment of amino acid adequacy. We situate dietary protein within the broader context of overall dietary patterns, emphasizing plant-rich diets and their relevance for long-term health outcomes in populations with overall adequate protein intake.

**Recent Findings:**

Recent evidence from randomized controlled trials and large-scale prospective cohort studies indicates that, although animal-derived protein sources typically achieve higher amino acid–based quality scores, these differences do not consistently translate into superior cardiometabolic or mortality-related outcomes. Substitution analyses and intervention studies suggest that replacing animal-based proteins with plant-based sources is associated with favorable changes in cardiovascular risk markers and reduced risk of chronic disease. These findings underscore the importance of the food matrix, including bioactive compounds, as well as the potential influence of components more prevalent in animal-based foods. In Western populations, where total protein intake often exceeds physiological requirements, modest differences in digestibility and amino acid scoring appear to have limited clinical relevance. Emerging evidence also suggest the may mediate host responses to different protein sources.

**Summary:**

Current evidence supports a context-dependent interpretation of protein quality that integrates amino acid adequacy within broader dietary and health frameworks. For clinical practice and dietary guidance, greater emphasis on overall dietary pattern quality, particularly plant-rich patterns, may be more informative for long-term health than reliance on protein quality scores alone.

**Graphical Abstract:**

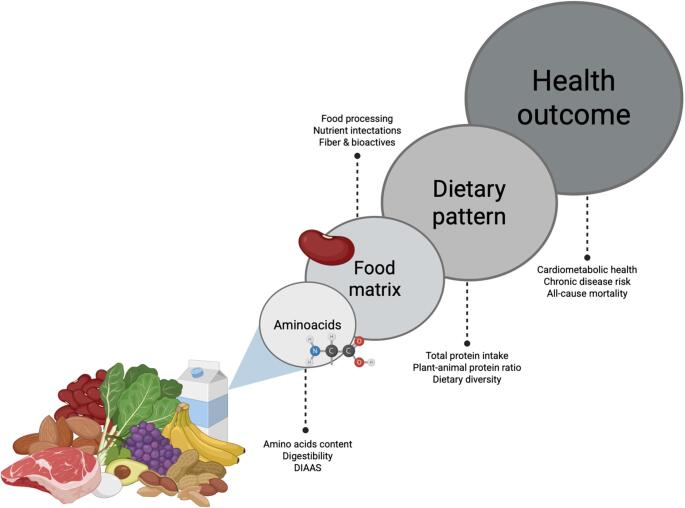

## Introduction

Dietary protein serves as the primary source of essential amino acids, which play a critical role in maintaining health and supporting physiological function. The concept of protein quality, defined as the capacity of a dietary protein to provide indispensable amino acids in proportions that meet metabolic requirements, has historically guided dietary recommendations. Animal-based protein sources are often considered higher quality when evaluated using conventional indices such as the protein efficiency ratio (PER), biological value (BV), and net protein utilization (NPU) [1]. However, these measures were developed under controlled experimental conditions and may not adequately capture the complexities of protein digestion, absorption, and metabolism within diverse real-world dietary patterns [2, 3].

More recently, advanced methods such as the Protein Digestibility Corrected Amino Acid Score (PDCAAS) and the Digestible Indispensable Amino Acid Score (DIAAS) have been developed to provide refined estimates of protein quality [4]. DIAAS is considered the reference method by the Food and Agriculture Organization (FAO) because it overcomes several limitations of PDCAAS, providing a more accurate assessment of amino acids absorbed by the human body and avoiding the overestimation inherent in fecal-based methods. Unlike PDCAAS, DIAAS scores are not capped at 1.0, enabling quantitative comparisons between proteins and revealing their capacity to complement other proteins in mixed diets [5]. By assessing ileal amino acid digestibility, DIAAS shows that animal-based protein sources such as egg, pork, and whey typically achieve values above 100, a threshold considered indicative of high-quality protein [4, 6]. Most plant-based protein sources score lower, with notable exceptions including soy, potato, and certain plant-based meat alternatives, which can reach DIAAS values comparable to animal-based proteins [7].

However, several limitations have been raised with the DIAAS and should be noted. As outlined by Craddock et al. (2021), DIAAS applies a single nitrogen to protein conversion factor of 6.25 instead of using food specific factors [8]. This approach tends to underestimate DIAAS values for many plant proteins and overestimate those of animal proteins, which is consistent with evidence on variation in nitrogen conversion factors across foods [9]. Most DIAAS values are derived from animal models, primarily pigs, which have been extensively validated for estimating ileal amino acid digestibility, although they may not capture all aspects of human digestion under free-living dietary conditions [10–13]. Finally, DIAAS is established for individual foods and does not account for the effects of food combinations within mixed meals, thereby not capturing potential interactions among foods that may influence overall amino acid availability and utilization in habitual diets [14].

Despite the technical nature of protein quality scoring systems, their terminology has strongly influenced how protein rich foods are perceived. The longstanding classification of animal proteins as “high quality” and plant proteins as “lower quality” has contributed to the widespread assumption that animal derived proteins are inherently superior, an idea reinforced by traditional amino acid based scoring frameworks that feature heavily in nutrition education and dietary guidance [14, 15]. Studies of consumer perception further show that people commonly interpret these terms as general markers of nutritional superiority, which contributes to the view that plant proteins are less adequate even when total protein intake is unlikely to be limiting in typical Western diets [16]. Moreover, Katz et al. (2019) note the conventional framing of protein quality can be easily misunderstood outside its scientific context, encouraging assumptions that higher amino acid scores translate directly into better health outcomes [17]. These interpretations persist even though protein quality metrics were not designed to predict long term health and do not reflect the broader dietary patterns in which proteins are consumed.

Building on the distinction between protein quality scoring systems and real-world health outcomes, evidence from epidemiological studies and randomized controlled trials suggests that dietary patterns differing in protein quality scores can be associated with similar or divergent long-term health outcomes. This observation raises an important methodological question in nutrition science: to what extent do protein quality metrics inform the interpretation of health effects within whole dietary patterns? Accordingly, this review examines the role and limitations of protein quality metrics when applied beyond amino acid adequacy and proposes a context-dependent framework for evaluating dietary protein in relation to human health.

## Rethinking Protein Quality: Implications for Muscle Health and Function

Protein quality has traditionally been considered a key determinant of functional outcomes related to dietary protein intake, including muscle mass and strength. Although many dietary plant protein sources score lower on established protein quality metrics, this does not appear to compromise these functional outcomes. Evidence from dietary patterns relying exclusively on plant-based protein sources, such as vegan diets, shows that gains in lean body mass and strength performance can be comparable to those observed in omnivorous diets when total protein and energy intake are matched [18]. An important consideration when interpreting evidence on protein quality is its interaction with total protein intake. Differences in protein quality are most relevant when protein intakes are close to physiological requirements, whereas at higher intakes their practical significance diminishes. In many randomized controlled trials examining muscle mass, strength, and metabolic outcomes, total protein intakes typically range between approximately 1.2 and 1.6 g·kg⁻¹·day⁻¹, well above the recommended dietary allowance [19–21]. Under these conditions, total indispensable amino acid requirements are readily met, reducing the likelihood that modest differences in amino acid digestibility or composition translate into meaningful functional differences. Moreover, it is important to note that, in athletic populations, exercise enhances nitrogen retention by improving the efficiency of dietary protein utilization [22]. Through adaptations to mechanical tension, metabolic stress, and muscle repair signaling, trained individuals retain a greater proportion of dietary nitrogen, such that increases in training load enhance protein utilization efficiency rather than requiring substantially higher absolute protein intakes [23, 24].

In older adults, a population particularly vulnerable to anabolic resistance and sarcopenia, consuming a variety of plant-based proteins within an isocaloric, isonitrogenous vegan diet supports muscle protein synthesis at rates similar to those of omnivorous diets [25, 26]. These findings suggest that plant proteins can effectively maintain muscle mass even in the context of age-related declines in anabolic responsiveness. Collectively, this evidence indicates that for outcomes such as muscle maintenance and sarcopenia prevention, the total amount and diversity of amino acids consumed may be more important than protein quality scores alone [27], demonstrating that proteins traditionally considered “lower quality” can adequately sustain muscle function across the lifespan.

## Protein Quality and Health Outcomes: Evidence for a Paradox

Historically, protein quality has been defined, operationalized, and communicated within scientific, regulatory, and dietary contexts, primarily to assess amino acid adequacy in relation to physiological requirements. Authoritative bodies, including the Food and Agriculture Organization (FAO) and World Health Organization (WHO), have addressed protein quality within broader nutritional approaches focused on growth, maintenance, and functional outcomes, rather than as a direct predictor of long-term disease risk [28, 29]. In this context, the 2013 FAO report associated protein quality with numerous physiological and clinical outcomes, including cardiovascular disease, cancer, hypertension, oxidative stress, aging, linear growth, cognitive function, immunity, and tissue repair, implying that higher-quality proteins confer superior health effects [29]. Within these frameworks, plant-derived proteins were often characterized as having lower protein quality on the basis of amino acid composition and digestibility, leading to recommendations focused on protein complementation or increased intake to ensure adequacy. Although these approaches were not intended to evaluate the overall healthfulness of foods or dietary patterns, their framing has contributed to the broader perception that animal-derived proteins are inherently superior for human health, whereas plant-based proteins are nutritionally inadequate unless specifically balanced.

Although animal-based protein sources score higher on established protein quality metrics such as DIAAS, they do not consistently confer superior health outcomes (Table 1). In fact, a recent network meta-analysis of randomized controlled trials found that replacing red meat, a protein source with a high DIAAS score, with plant-based protein sources was associated with favorable changes in several cardiovascular risk factors, including total cholesterol and low-density lipoprotein cholesterol (LDL-C) [32]. Large epidemiologic studies similarly show that isocaloric substitution of plant-based proteins, particularly legumes and nuts, for animal proteins such as red or processed meat is linked to lower risks of multiple non-communicable chronic diseases and all-cause mortality [33, 37, 38]. For example, replacing 3% of energy from animal protein with plant protein has been associated with reductions of approximately 10% in all-cause mortality and 11–12% in cardiovascular mortality, while substitutions of 5% have been linked to greater reductions, reaching 24% for all-cause mortality and 22% for cardiovascular mortality [39, 40]. Consistent with these findings, analyses of the Nurses’ Health Study (NHS), NHS II, and Health Professionals Follow-up Study cohorts further revealed that a higher plant-to-animal protein ratio, which may correspond to a lower average protein quality according to conventional metrics, was associated with reduced risks of cardiovascular disease and coronary artery disease [41].

**Table 1:** Comparison of protein quality (DIAAS) with health outcomes across protein sources

Source	Protein quality (DIAAS)	Surrogate markers (RCT evidence)	All-cause mortality (Epidemiologic evidence)
Red meat	111 [30]		
*vs. legumes*	83–103 [6, 31]	↓ total cholesterol and low-density lipoprotein cholesterol [32]	0.97 (0.92–1.03) [33]
*vs. nuts*	83 [34]		0.93 (0.91–0.95) [33]
*vs. whole grains*	68 [6]		0.96 (0.95–0.98) [33]
Dairy	123 [35]		
*vs. nuts/legumes*	83–103 [6, 31, 34]	↓ low-density lipoprotein cholesterol, and blood pressure [36]	0.96 (0.94–0.99) [33]
*vs. nuts*	83 [34]		0.94 (0.91–0.97) [33]
*vs. whole grains*	68 [6]		0.91 (0.85–0.97) [36]
Eggs	111 [6]	↓ low-density lipoprotein cholesterol, and high-density lipoprotein cholesterol [74, 75]	
*vs. legumes*	83–103 [6, 31]		0.85 (0.82–0.89) [33]
*vs. nuts*	83 [34]		0.90 (0.89–0.91) [33]

Protein quality data are expressed as mean individual DIAAS values. Representative DIAAS values correspond to the following foods: red meat (beef and pork), legumes (soy, beans, and peas), nuts (pistachios), and whole grains (oats). All-cause mortality is reported as summary hazard ratios (HRs) with 95% confidence intervals, derived from substitution meta-analyses examining the replacement of animal-based foods with plant-based foods

In light of this evidence, the scientfic report of the 2025 Dietary Guidelines Advisory Committee of the United States has prioritized plant-based proteins, especially legumes, over animal-based sources such as meat, poultry, and eggs [42, 43]. These findings challenge the traditional assumption that higher protein quality, as defined by amino acid composition and digestibility, automatically translates into better health outcomes. They suggest that the context in which protein is consumed, including the broader dietary pattern or the food matrix, plays a crucial role in modulating health effects. These observations support a more nuanced, holistic framework for evaluating dietary protein within the context of overall health.

## Beyond Protein: The Role of the Food Matrix in Health Outcomes

Resolving this apparent paradox requires acknowledging a fundamental principle of nutritional science: food components do not act in isolation, and the health effects of diets rich in plant-based foods reflect a dual influence [43]. First, such diets avoid potentially harmful components commonly found in animal-based matrices, including saturated fat, cholesterol and heme iron [44–46]. Higher intakes of saturated fat have been linked to activation of proinflammatory pathways such as interleukin-1 (IL-1), interleukin-6 (IL-6) and tumor necrosis factor-alpha (TNF-α) and are associated with elevated C reactive protein concentrations [47]. In addition, animal based diets tend to produce higher circulating concentrations of trimethylamine N-oxide (TMAO) due to gut microbial metabolism of carnitine and choline, compounds primarily found in animal products, with evidence linking TMAO to cardiovascular disease and low-grade systemic inflammation [48, 49]. Second, they provide beneficial compounds inherent to plant foods, such as dietary fiber and bioactive compounds including phytochemicals and antioxidants [17, 50]. These compounds have been shown to reduce oxidative stress, modulate inflammatory pathways and improve endothelial function, contributing to the more favourable inflammatory and cardiometabolic profiles observed in plant-based dietary patterns [51, 52].

This reflects a conceptual mismatch in which a single-factor metric, such as when DIAAS is used to predict a complex, multifactorial outcome like overall health [53]. For chronic disease prevention, focusing on a high-quality dietary patterns, rich in whole plant-based foods is more informative than concentrating solely on protein quality.

### The Gut-Protein-Health axis

The effects of dietary protein on human health are influenced not only by amino acid composition and digestibility but also by interactions with the gut microbiota. Plant-based protein sources are generally less digestible than animal-based protein sources, resulting in a greater proportion reaching the colon and undergoing microbial fermentation. Although protein fermentation can produce metabolites with potentially adverse effects [54, 55], higher intake of plant-based protein sources is consistently associated with favorable health outcomes [33, 56]. This apparent contradiction likely reflects the broader dietary context. Plant-based protein sources are typically consumed alongside fiber and other bioactive compounds that modulate microbial activity and promote host health [57, 58]. Notably, adjustments for dietary fiber intake in recent large cohort studies did not alter the beneficial associations of plant-based protein sources with health outcomes, suggesting that these benefits extend beyond those attributable to fiber alone [59, 60].

Plant-based protein sources promote the growth of Lactobacillus and Bifidobacterium and are associated with higher alpha diversity and enrichment of beneficial taxa such as Bacteroidetes, Prevotella, and Roseburia, a butyrate producer essential for gut health [61, 62]. In contrast, long-term consumption of animal-protein-rich diets has been linked to an increased abundance of Alistipes and Bacteroides and a reduction in Roseburia [61]. Moreover, certain components of animal-based protein sources, such as choline in red meat, can be metabolized by the microbiota into TMAO, which is associated with adverse health effects [63]. These findings highlight the important role of the gut microbiota in modulating the health effects of dietary protein and underscore the need for future studies to clarify these complex interactions.

## Protein Quality in Context

Given that total protein intake in Western populations generally exceeds established physiological requirements, an emphasis on protein quality alone may be misplaced, and greater attention may be warranted on the overall quality of dietary patterns. The estimated average requirement (EAR) for protein is 0.66 g per kilogram per day, while the recommended daily allowance (RDA) is 0.8 g per kilogram per day [64, 65]. Even among individuals consuming entirely plant-based diets, such as the EPIC Oxford cohort who averaged 0.99 g per kilogram per day, they surpassed the recommended intake by approximately 24% [8]. At this intake level, meeting the FAO WHO UNU requirement for total indispensable amino acids (0.184 g per kilogram per day) equates to roughly 19% of total protein [19]. This requirement can be met by the intrinsic amino acid composition of common plant foods, which contain an average of 26% indispensable amino acids, with relatively little variation across staples such as oats, wheat, rice, peas, corn and potatoes [66], a proportion that exceeds the reduced indispensable amino acid requirement at typical intake levels [67]. Consistent with this, a meta analysis of nitrogen balance studies reported no significant differences in protein requirements among diets dominated by plant protein, animal protein or mixed protein sources, indicating that overall protein intake rather than protein quality is the primary determinant of adequacy in healthy adults [68]. Within this context, modest differences in protein quality indices have limited nutritional relevance for most individuals in Western settings. Previous studies have suggested that protein quality metrics such as DIAAS are most relevant in contexts of protein energy malnutrition and food insecurity, and that in developed nations, emphasis on protein quality may shift attention away from more impactful dietary priorities including fibre intake and overall dietary pattern quality, which are likely to exert far greater influence on long term health outcomes [53].

## Conclusions

Although animal-based protein sources generally score higher on established protein quality metrics, available evidence indicates that plant-based protein sources can support comparable functional outcomes and favorable long-term health profiles when consumed within appropriate dietary contexts. Protein quality captures an important but limited aspect of nutritional adequacy and should not be interpreted as a proxy for the overall healthfulness of foods or dietary patterns. In populations with adequate protein intake, modest differences in protein digestibility or amino acid scoring are therefore unlikely to translate into meaningful differences in long-term health outcomes, underscoring the need to evaluate dietary protein within whole dietary patterns rather than in isolation.

## Key References

- Craddock, J.C.; Genoni, A.; Strutt, E.F.; et al. Limitations with the Digestible Indispensable Amino Acid Score (DIAAS) with Special Attention to Plant-Based Diets: A Review. *Current Nutrition Reports* 2021, 10, 93–98, doi:10.1007/S13668-020-00348-8.
  - ⚬ This review critically examines the methodological limitations of DIAAS, including the use of a single nitrogen-to-protein conversion factor and reliance on animal models, and discusses how these factors may bias the evaluation of plant-based protein sources.
- Katz, D.L.; Doughty, K.N.; Geagan, K.; et al. Perspective: The Public Health Case for Modernizing the Definition of Protein Quality. *Advances in Nutrition* 2019, 10, 755–764, doi:10.1093/advances/nmz023.
  - ⚬ This perspective article argues for a broader, public health–oriented definition of protein quality that extends beyond amino acid adequacy to incorporate long-term health outcomes and dietary pattern context.
- Glenn, A.J.; Wang, F.; Tessier, A.-J.; et al. Dietary Plant-to-Animal Protein Ratio and Risk of Cardiovascular Disease in Three Prospective Cohorts. *American Journal of Clinical Nutrition* 2024, 120, 1373–1386, doi:10.1016/j.ajcnut.2024.09.006.
  - ⚬ This large prospective cohort analysis links higher plant-to-animal protein ratios to lower cardiovascular disease risk, supporting the relevance of protein source and dietary patterns over amino acid–based quality scores alone.

## Data Availability

Not applicable.
